# Transcortical sensory aphasia heralding SARS-Cov-2-induced autoimmune encephalitis with gyral restricted diffusion hyperintensities: a novel case report

**DOI:** 10.1186/s41983-022-00593-4

**Published:** 2022-12-29

**Authors:** Arka Prava Chakraborty, Alak Pandit, Ajitava Dutta, Shambaditya Das, Goutam Ganguly, Souvik Dubey

**Affiliations:** Department of Neurology, Bangur Institute of Neurosciences, 52/1A SN Pandit Street, Kolkata, West Bengal India

**Keywords:** COVID-19, COVID-19 autoimmune encephalitis, Cognitive dysfunction, Gyral restricted diffusion hyperintensities, Transcortical sensory aphasia

## Abstract

**Background:**

Coronavirus disease 2019 (CoVID-19), primarily thought of as a respiratory system disease is actually a multi-system disease with immunological implications. CNS involvement in COVID has been explained in recent literature mainly for stroke, encephalopathy, encephalitis, acute disseminated encephalomyelitis and myelopathy. There are few studies characterizing clinical spectrum of COVID autoimmune encephalitis. We present a unique case of post-COVID autoimmune encephalitis in a diabetic male presenting with language dysfunction and novel radiologic findings.

**Case presentation:**

Patient admitted to inpatient department of a tertiary care hospital of India was evaluated by bedside clinical examination, routine blood tests, CSF study with intrathecal SARS-Cov-2 antibody detection, commercially available tests for autoimmune encephalitis, neuroviral panel with HSV PCR, EEG, 3-Tesla MRI and PET scan. Patient was found to have personality change and transcortical sensory aphasia in the outset of COVID encephalitis. MRI findings like temporal involvement and insular ribboning are also being reported. The patient was treated with IV immunoglobulin and is on an improving course.

**Conclusions:**

This case reports dysphasia due to COVID-mediated injury to the language networks, with novel radiologic findings. Role of parainfectious versus immune etiology is also discussed. Further studies are needed to elucidate the mechanism and clinical spectrum of post-COVID autoimmune encephalitis.

## Background

Encephalopathy in novel coronavirus disease 2019 (COVID-19) has been demonstrated in literature to be due to hypoxic damage, metabolic abnormality, stroke, encephalitis and acute disseminated encephalomyelitis (ADEM) [5]. COVID encephalitis has been described mainly in various case reports and reviews without concrete characterization of clinical, cognitive and radiologic spectrum. Autoimmune encephalitis (AE) following preceding viral infection has been well encountered in the past. Similar reports of AE have also emerged following CoVID-19 infection at a frequency amounting up to 5 per ~ 10,000 COVID hospitalized patients [2]. Controversy lies between direct viral entry and immune-mediated mechanism for central nervous system involvement. Here, we present an interesting case of COVID-19 autoimmune encephalitis presenting with language dysfunction and novel radiologic findings.

## Case presentation

A 61-year-old hypertensive and diabetic unvaccinated male, had one episode of mild fever and dry cough at day 1 of illness. Patient tested COVID RT PCR positive on Day 2 of fever and was kept in home isolation. On Day 5, patient developed abnormal behavior, started speaking irrelevantly and then gibberish (as told by patient’s wife) and became apathetic with progressive drowsiness, when he was admitted to primary care on Day 9. Patient was evaluated and found to have hyponatremia without COVID pneumonia or cytokine storm. Hyponatremia was corrected as per protocol, however there was no expected improvement in patient’s behavioral abnormality. Patient was referred to us for further evaluation on Day 15.

**Fig. 1:**
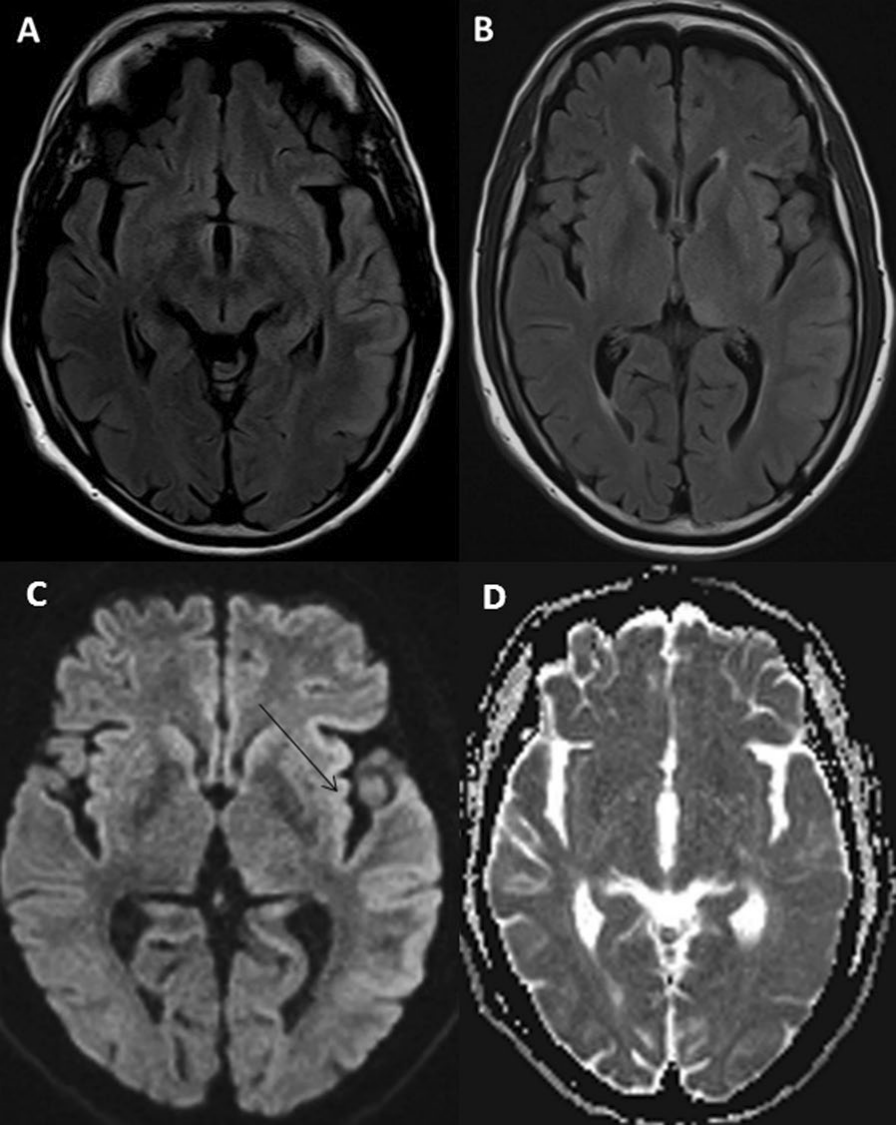
**A** and **B** Magnetic resonance imaging T2-weighted image shows hyperintensities in left temporal lobe, insula and left thalamus. **C** and **D** Diffusion restriction with normal apparent diffusion coefficient. Black arrow shows “insular ribboning”

**Fig. 2:**
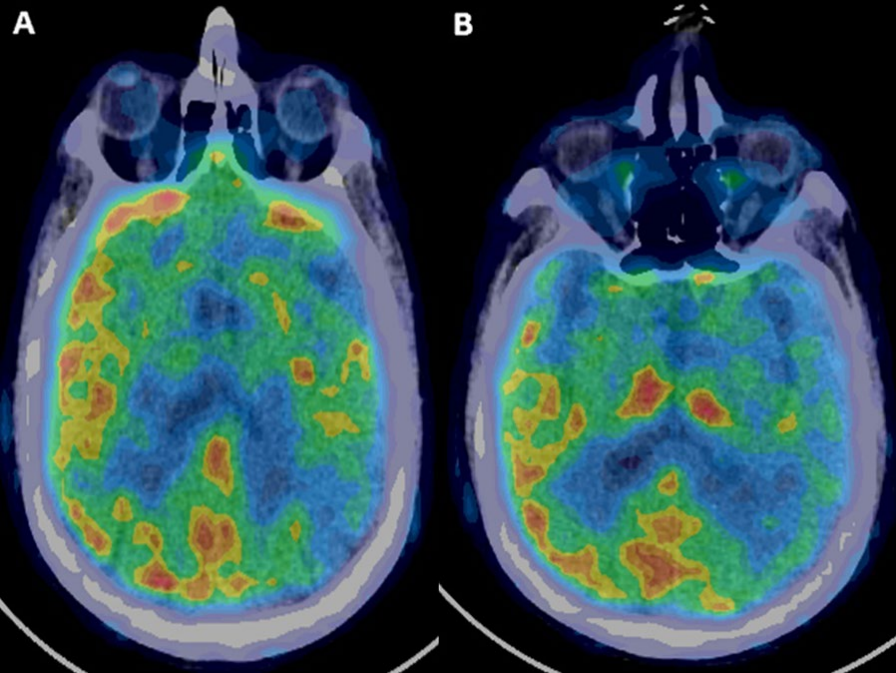
Positron emission tomography (PET) with ^18^fluorodeoxyglucose shows hypometabolism in left temporal lobe and adjacent parietal and occipital lobes on ^18^fluorodeoxyglucose positron emission tomography (FDG PET)

Examination revealed low mood, language dysfunction in the form of impaired comprehension, paraphasias, jargons, circumlocutions, perseveration with preserved fluency, grammar and repetition. Both alexia and agraphia were present. Language dysfunction was concluded as transcortical sensory aphasia. Rest of cognitive examination could not be done due to comprehension deficit. Motor, sensory, autonomic and systemic examination was normal. MRI brain revealed T2-hyperintense lesions in gyral distribution of left temporo-parietal regions and left thalamus with temporal cortical and insular ribboning with diffusion restriction with normal apparent diffusion coefficient (Fig. 1). PET showed severe hypometabolism in left temporal, parieto-occipital and basifrontal regions (Fig. 2). Electroencephalogram was normal. CSF study showed elevated protein (66 mg/dl), with negative CSF CoVID-19 RT PCR but detectable SARS-Cov-2 IgG and spike protein antibodies. CSF Herpes Simplex virus (HSV) 1 and 2 PCR, autoimmune encephalitis panel (including NMDA-R, GABA-A/B, AMPA, LGI2 AND CaSPR2 antibodies) and paraneoplastic panel were negative. Paraneoplastic workup was normal. Patient fulfilled diagnostic criteria of possible autoimmune encephalitis [6] and was given a course of intravenous methylprednisolone followed by intravenous immunoglobulin (IVIG) at 2 g/kg divided over 5 days. Currently, patient is on an improving course. There is marked improvement in language function seen during follow-up at 3 months.

Diagnosis of COVID-19-related possible autoimmune encephalitis in our patient can be ascertained by fulfillment of the Graus 2016 criteria [6], the onset of symptoms after four days of testing positive for COVID RT PCR (99% specificity), radiologic evidence of encephalitis with corresponding hypometabolism in PET–CT and evidence of intrathecal SARS Cov-2 IgG and spike protein IgG antibodies [3]. HSV encephalitis, a close differential usually presents with seizures, behavioral abnormality, unconsciousness or confusional state. Speech disturbances are reported in 57% patients [14]. Normal CSF study, negative HSV PCR and improvement by IVIG without acyclovir are arguments against HSVE. Absence of central pontine lesion and basal ganglia involvement, presence of language dysfunction and the lack of extrapyramidal features weighs against the possibility of osmotic demyelination occurring during hyponatremia correction. Moreover, the presence of clinical problem even after correction of hyponatremia points towards non-metabolic causes. Other differentials like autoimmune and paraneoplastic encephalitis due to membrane or intracellular antibodies were also ruled out. Other major causes of post-COVID-19 encephalopathy described in current literature are ADEM and acute hemorrhagic leukoencephalopathy (AHLE). They are reported in a systemic review of 46 patients [10], with 2/3rd having ADEM and others AHLE. Most of them followed severe Covid infection and had poor outcomes with immunomodulation with 32% mortality and ≥ 4 modified Rankin score in 64% patients. Most of the patients had white matter periventricular lesions on MRI. Clinical and radiologic phenotype of our patient was much more different and pointed mainly towards grey matter pathology. Absence of seizure in our patient with normal EEG also rules out the radiological differential of post-ictal change.

Post-COVID autoimmune encephalitis may occur after few days following onset of respiratory symptoms of COVID-19 or may present as fever with subacutely developing encephalopathy [2]. In the cohort of Mayo Clinic, Minnesota the reported median age of presentation was 61 years and all of the patients were above the age of 45. Involvement ranged from a normal imaging to bilateral medial temporal, rhombencephalitis, cerebellar and pontine involvement. Another case described limbic encephalitis in a 74-year-old female with altered sensorium and left temporal onset focal motor seizures, 2 weeks after COVID infection [13].

MRI abnormalities reported in COVID patients are nonspecific T2 cortical and subcortical hyperintensities, microbleeds, hemorrhagic thalamic lesions, hippocampal and mesial temporal lobe hyperintensities, brainstem encephalitis, posterior reversible leukoencephalopathy, acute necrotizing encephalopathy and ADEM-like lesions [3, 5]. In another study, brain MRI abnormalities were found in 37% of patients with cortical diffusion restriction and low ADC signal mainly in the frontal and occipital lobes with sparing of deep grey matter [7]. We report left insular ribboning with temporal, basifrontal and thalamic changes in our patient. This affliction involving major centers of the language network was expected to produce major deficits. Clinically, as per the findings above, our patient was concluded to have transcortical sensory aphasia.

Apart from stroke, language dysfunction in the form of expressive aphasia has been shown in patients with COVID encephalopathy with normal MRI and electroencephalogram [11, 12]. In these patients, there were no radiologic or CSF evidence of encephalitis. Patients showed good response to immunotherapy. A frontal dysexecutive syndrome comprising inattention, disorientation and impaired concentration has been reported in 36% of the patients presenting with CNS COVID involvement [8]. Alexia and agraphia can occur as an isolated syndrome or in presence of aphasia. Anatomical substrates implicated for agraphia are the dominant angular gyrus with its connections and in some cases the lateral occipital gyri [15]. Presence of gross sensory aphasia and the affliction of the left perisylvian language areas, lateral temporal and occipital lobes very well explains the presence of both alexia and agraphia in our patient [9]. Our patient had initial apathy, improving later with patient becoming overtly meek and gentle than premorbid state, with repeated gesturing with folded hands and requested to go home. Executive dysfunction was not marked, as he could carry out his own works like eating, bathing, dressing without help.

Most studies have found negative CSF RTPCR for COVID, but the presence of intrathecal IgG SARS-Cov-2 antibodies in CNS COVID are much more frequent [3]. Though our patient had temporal relation supporting parainfectious or direct neuroinvasion theory, literature backs the concept of immune-mediated injury [4]. Molecular mimicry with SARS-Cov-2 antigens may trigger CNS autoantibodies. Neuropathology and immunofluorescence studies of COVID-19 infected brain have shown patterns consistent with autoimmune encephalitides. SARS-Cov-2 can mount a chronic immune-mediated process in the CNS by triggering type I interferons [1]. They may also lead to α-synuclein aggregation and further TLR4, 6 and 8 mediated CNS damage. IL-10 on the other hand has been found to have anti-inflammatory effects and checks the immune-mediated damage to the CNS. As with other post-infective phenomenon like post HSVE NMDAr encephalitis, SARS-Cov-2 may trigger a “long-CoVID” syndrome following initial infection, by immune-mediated injury [4]. Moreover, immunotherapy with plasmapheresis, steroids or IVIG have shown great results in CNS involvement of CoVID.

## Conclusions

To conclude, language dysfunction, novel radiologic findings and good response to immunotherapy, make this case a gem of clinical neuroimmunology in the current context of COVID-19.

## Data Availability

The authors declare and confirm that the data supporting the findings are available in this manuscript and that it can be shared openly, including deposition in a repository, as per the journal’s preference.

## Abbreviations

COVID-19: Novel coronavirus disease 2019
ADEM: Acute disseminated encephalomyelitis
AE: Autoimmune encephalitis
RT PCR: Real-time polymerase chain reaction
CSF: Cerebrospinal fluid
GABA: Gamma aminobutyric acid
NMDA: N-Methyl D aspartate
LGI2: Leucine rich glioma inactivated protein
AMPA: α-Amino-3-hydroxy-5-methyl-4-isoxazolepropionic acid
CASPR-2: Contactin-associated surface protein receptor type 2
HSV: Herpes simplex virus
PET: Positron emission tomography
IVIG: Intravenous immunoglobulin
